# Supplementary material for: Controlling the Oxidation of Magnetic and Electrically Conductive Solid-Solution Iron-Rhodium Nanoparticles Synthesized by Laser Ablation in Liquids
Source: Nanomaterials (Basel). 2020 Nov 27;10(12):2362. doi: 10.3390/nano10122362 (PMC7760681; doi:10.3390/nano10122362)
Supplement: Supplementary file 1 [file nanomaterials-10-02362-s001.pdf]

Supplementary materials
Controlling the Oxidation of Magnetic and Electrically Conductive Solid-Solution Iron-Rhodium Nanoparticles Synthesized by Laser Ablation in Liquids

Ruksan Nadarajah [1], Shabbir Tahir [1], Joachim Landers [2], David Koch [3], Anna S. Semisalova [2], Jonas Wiemeler [2], Ayman El-Zoka [4], Se-Ho Kim [4], Detlef Utzat [2], Rolf Möller [2], Baptiste Gault [4,5], Heiko Wende [2], Michael Farle [2] and Bilal Gökce [1,*]

[1] Technical Chemistry I and Center for Nanointegration Duisburg-Essen (CENIDE), University of Duisburg-Essen, Universitaetsstr. 7, 45141 Essen, Germany; ruksan.nadarajah@uni-due.de (R.N.); shabbir.tahir@uni-due.de (S.T.); bilal.goekce@uni-due.de (B.G.)

[2] Faculty of Physics and Center for Nanointegration Duisburg-Essen (CENIDE), University of Duisburg-Essen, Lotharstr. 1, 47057 Duisburg, Germany; joachim.landers@uni-due.de (J.L.); anna.semisalova@uni-due.de (A.S.S.); jonas.wiemeler@uni-due.de (J.W.); detlef.utzat@uni-due.de (D.U.); rolf.moeller@uni-due.de (R.M.); heiko.wende@uni-due.de (H.W.); michael.farle@uni-due.de (M.F.)

[3] Institute of Materials Science, University of Technology, Darmstadt 64287, Alarich-Weiss-Strasse 2, Germany; david.koch@tu-darmstadt.de

[4] Max-Planck-Institut für Eisenforschung GmbH, Max-Planck-Strasse 1, 40237 Düsseldorf, Germany; a.elzoka@mpie.de (A.E.-Z.); s.kim@mpie.de (S.-H.K.); b.gault@mpie.de (B.G.)

[5] Department of Materials, Royal School of Mines, Imperial College London, London SW7 2AZ, UK

* Correspondence: bilal.goekce@uni-due.de; Tel.: +49-201-183-3146

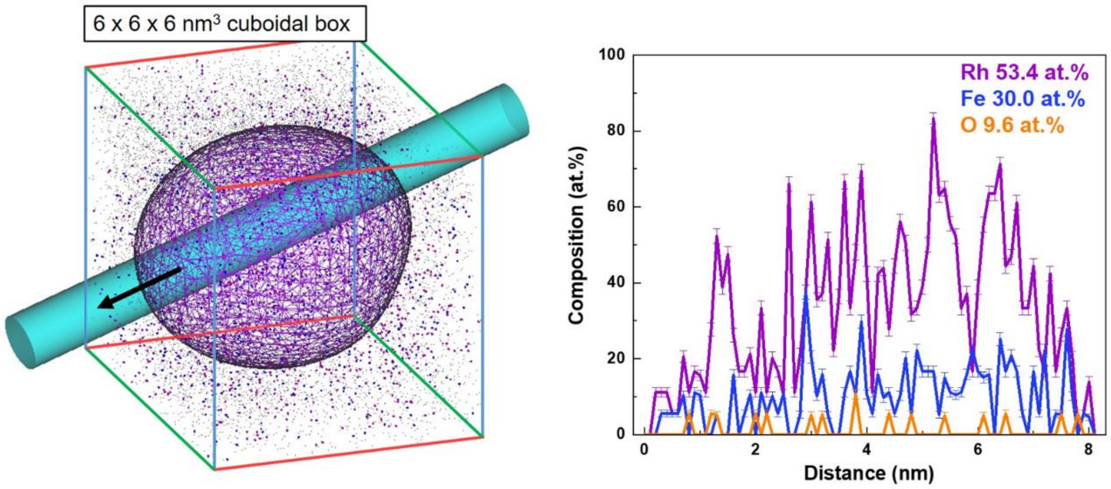

Figure S1. Atom probe tomography of FeRh nanoparticles synthesized in acetone and argon atmosphere. Extracted FeRh nanoparticle (25 at% of FeRh iso-composition surface) and line scan of selected FeRh nanoparticle.
